# Genome structure reveals the diversity of mating mechanisms in *Saccharomyces cerevisiae* x *Saccharomyces kudriavzevii* hybrids, and the genomic instability that promotes phenotypic diversity

**DOI:** 10.1099/mgen.0.000333

**Published:** 2020-02-17

**Authors:** Miguel Morard, Yaiza Benavent-Gil, Guadalupe Ortiz-Tovar, Laura Pérez-Través, Amparo Querol, Christina Toft, Eladio Barrio

**Affiliations:** ^1^​ Departament de Genètica, Universitat de València, Burjassot, Valencia, Spain; ^2^​ Departamento de Biotecnología de los Alimentos, Instituto de Agroquímica y Tecnología de los Alimentos (IATA), CSIC, Paterna, Valencia, Spain; ^†^​ Present address: Centro de Estudios Vitivinícolas de Baja California, México, CETYS Universidad, Ensenada, Baja California, Mexico; ^‡^​ Present address: Institute for Integrative and Systems Biology, Universitat de València and CSIC, Paterna, Valencia, Spain

**Keywords:** *Saccharomyces cerevisiae*, *Saccharomyces kudriavzevii*x, hybrids, hybridization mechanisms, rare-mating, genome rearrangements

## Abstract

Interspecific hybridization has played an important role in the evolution of eukaryotic organisms by favouring genetic interchange between divergent lineages to generate new phenotypic diversity involved in the adaptation to new environments. This way, hybridization between *Saccharomyces* species, involving the fusion between their metabolic capabilities, is a recurrent adaptive strategy in industrial environments. In the present study, whole-genome sequences of natural hybrids between *Saccharomyces cerevisiae* and *Saccharomyces kudriavzevii* were obtained to unveil the mechanisms involved in the origin and evolution of hybrids, as well as the ecological and geographic contexts in which spontaneous hybridization and hybrid persistence take place. Although *Saccharomyces* species can mate using different mechanisms, we concluded that rare-mating is the most commonly used, but other mechanisms were also observed in specific hybrids. The preponderance of rare-mating was confirmed by performing artificial hybridization experiments. The mechanism used to mate determines the genomic structure of the hybrid and its final evolutionary outcome. The evolution and adaptability of the hybrids are triggered by genomic instability, resulting in a wide diversity of genomic rearrangements. Some of these rearrangements could be adaptive under the stressful conditions of the industrial environment.

## Data Summary

All the genomic sequence data of the natural and artificial *S. cerevisiae* x *S. kudriavzevii* hybrids has been previously uploaded to the National Center for Biotechnology Information (NCBI) GenBank under the BioProject accession number PRJNA531118. Genome sequences of reference *S. cerevisiae* and *S. kudriavzevii* strains were retrieved from previous studies and downloaded from NCBI, *Saccharomyces* Genome Database (SGD, https://www.yeastgenome.org), *Saccharomyces* Resequencing Genome Project (SRGP, http://www.sanger.ac.uk/research/projects/genomeinformatics/sgrp.html) and European Nucleotide Archive (ENA) databases, as indicated in Table S1, available in the online version of this article. All software used in the analyses of the genome sequences are publicly available, and the sources have been provided within the article.

**Impact Statement**

In the last decade, a great effort has been devoted to the study of natural *Saccharomyces* hybrids present in industrial fermentations. These hybrids originated by spontaneous hybridization between *S. cerevisiae* and a cryophilic species of this genus, such as *S. eubayanus* (lager beer), *S. kudriavzevii* (ale beer and wine) or *S. uvarum* (wine and cider) [13, 23]. The characterization of natural hybrids demonstrated that they inherited the good fermentation performance and ethanol tolerance of *S. cerevisiae* and the ability to grow at lower temperatures of the cryophilic partner, as well as other properties of interest. This prompted the development of artificial hybrids for industrial applications, which are usually obtained by ‘canonical’ mating between haploid spores. However, the frequent triploidy of natural hybrids indicates that ‘rare’ mating could be a probable mechanism of hybridization. As the genomic architectures of hybrids will differ depending on the mating, the deciphering of the hybridization mechanisms involved in the origin of natural hybrids, which is the purpose of this study, is critical to design new breeding programs of industrial yeasts through hybridization. Moreover, this study also contributes to understanding how hybridization generates genome instability and variability that, under the selective pressures present in fermentation environments, can generate functional innovation.

## Introduction

Hybridization, considered as reproduction between individuals belonging to genetically distinct populations or different species [1], has played an important role in the evolution of many eukaryotic organisms. This way, the genetic interchange between divergent lineages, due to hybridization, can generate new phenotypic diversity through the expression of hybrid vigour, allows for adaptation to new environments through the introgression of novel alleles and transgressive segregation [2], and may contribute to the formation of new hybrid species [3], either by allopolyploidy, when the ploidy of hybrids increases with respect to that of the parental species, or homoploidy, when ploidy remains unaltered [4].

Hybridization and its evolutionary consequences in speciation and adaptation have been widely studied in plants [5–7] and animals [3, 8, 9], including hominins [10], but not so extensively in fungi. In fungi, attention was mainly focused on hybridization in pathogenic fungi [11, 12] and yeasts of biotechnological interest [13, 14], being hybrids of the *Saccharomyces* genus the most studied examples [15, 16], including the role of hybridization in yeast speciation and adaptation [17–22].

At present, the *Saccharomyces* genus is composed of eight species: *S. arboricola, S. cerevisiae*, *S. eubayanus*, *S. jurei, S. kudriavzevii*, *S. mikatae, S. paradoxus* and *S. uvarum* [23–25]. These species show postzygotic reproductive isolation [26], and therefore, mating between them is possible and hybridization is easily achieved in the laboratory [27, 28]. Some studies also demonstrated that interspecific hybridization can also occur in nature, in the insect gut [29, 30]. These interspecific hybrids are sterile, mainly due to nucleotide divergence that prevents spore viability [26, 31]. However, they are viable and can reproduce asexually by budding [15, 28].

The first and well-known example of interspecific *Saccharomyces* hybrid is the lager yeasts *S. pastorianus* (syn. *S. carlsbergensis*) [32], which is a partial allotetraploid hybrid between *S. cerevisiae* and another species, later identified as *S. eubayanus* [33]. Most natural hybrids have been isolated from fermentative environments in European regions with Continental and Oceanic climates, and they were generated by spontaneous hybridization between *S. cerevisiae* (*Sc*) and a cryophilic species: *S. eubayanus* (*Se*), *S. kudriavzevii* (*Sk*) and *S. uvarum* (*Su*) [13, 23].

Contrastingly, natural hybrids seem to be almost absent in wild environments, where only a few hybrids between the closely related species *Sc* and *S. paradoxus* (*Sp*) have been isolated [34]. However, the presence of introgressed nuclear genome regions between *Sc* and *Sp* [31, 35–37], as well as between *Se* and *Su* [38], another pair of closely related species, suggests hybridization occurs in the wild between closely related species but gives rise to unstable hybrids [16, 39]. This is also confirmed by the presence of introgressions in the mitochondrial genome of different *Saccharomyces* species [40].

The physiological characterization of industrial *Saccharomyces* hybrids demonstrated that they inherited the good fermentation performance of the *Sc* parent and the capability to grow at lower temperatures of the non-*Sc* partner, in addition to other properties of biotechnological interest [41–46]. These interesting properties contributed by the non-*Sc* species prompted the development of artificial interspecific hybrids for industrial applications [27, 47–50].

For more than one decade, our laboratory described and characterized, both at the molecular and physiological level, *Sc* x *Sk* hybrids (as reviews see [1, 50]). By combining the comparative genome characterization of hybrids, deduced from microarray hybridization [51], with a multilocus phylogenetic analysis [52], seven potential hybridization events were predicted as the origin of *Sc* x *Sk* hybrids [52], including the two most frequent hybrid lineages. One was predominant in Wädenswill, Switzerland, and was related to Trappist brewing hybrids, and the other was widely distributed from the Rhine valley (Alsace and Germany) to the Danube valley (Pannonian region: Austria, Croatia and Hungary).

In the present study, we selected different hybrid strains as representatives of the different groups defined according to those previous studies, to obtain whole-genome sequences to unveil the mechanisms involved in the origin and evolution of these hybrids, as well as the ecological and geographic contexts in which spontaneous hybridization and hybrid persistence take place. The understanding of the mechanisms involved in hybrid formation is also of interest to develop programs of industrial yeast improving based on artificial hybridization.

## Methods

### Strains and genome sequencing

In this study, we selected different hybrid strains (Table 1) as representatives of the different groups defined according to previous characterizations [51, 52]. The total yeast DNA extraction was performed according to the method described by Querol *et al*. [53]. Natural hybrids were sequenced with paired-end libraries of 100 bp with a mean insert size of 300 bp in an Illumina HiSeq 2500 instrument. The artificial *Sc* x *Sk* hybrid obtained (see below) was sequenced with ABI SOLiD paired-end of 25–75 nucleotides. Genome sequencing reads and assemblies of reference *Sc* T73 and *Sk* CR85 strains were obtained in previous studies [54, 55].

**Table 1.** Ploidy and spore viability of *S. cerevisiae* x *S. kudriavzevii* hybrids. Spore viability is expressed in percentage followed by the number of viable spores/total number of spores tested between brackets. nd, no data available because the number of asci was very small or absent. Ploidies were estimated in previous studies [51, 52]

Hybrids	Isolation source	Origin	Ploidy	Spore viability
VIN7	Wine	Alsace, France	3.07±0.08	7.81 % (5/64)
W27	Wine	Wädenswil, Switzerland	3.18±0.08	nd
IF6	Dietary complement	Barcelona, Spain	3.25	0 % (0/64)
CECT11002	Trappist beer	Louvaine-la-Neuve, Belgium	3.02±0.14	nd
MR25	Respiratory tract	Barcelona, Spain	2.92	10.94 % (7/64)
AMH	Wine	Geisenheim, Germany	3.85±0.18	nd
PB7	Wine	León, Spain	3.96±0.08	95.30 % (61/64)

### Genome assembly and annotation

The hybrid-genome sequence reads were trimmed with Sickle v1.2 [56] using a minimum quality per base of 28 and filtered with a minimum read length of 85nt. Velvet v1.2.03 [57] was used to determine which k-mer size was optimum for each sequencing library. The assembly step was performed with Sopra v1.4.6 [58] integrated with Velvet, by using the k-mer size determined previously. sspace v2.0 [59] and GapFiller v1.11 [60] were used to improve scaffold length and remove internal gaps. The resulting scaffolds were then aligned to a concatenated *Sc–Sk* genome reference with MUMmer v3.07 [61]. The genomes used as references were *Sc* T73 and *Sk* CR85. After the alignment, the scaffolds were organized into chromosomes with an in-house script.

Hybrid genomes were annotated using ratt [62] to transfer the annotation by sequence homology using the *Sc* T73 and *Sk* CR85 genome annotations. augustus web server [63] was used to complete the annotation in regions in which no gene transfer was obtained with ratt. Annotations were manually checked and corrected using Artemis [64].

### Mappings, variants detection and ratio analysis

Hybrid-genome mappings were performed against a concatenated reference of *Sc* T73 and *Sk* CR85. Illumina sequences were mapped by using bowtie2 v2.3.0 [65], with default parameters. SOLiD reads of the artificial hybrid were mapped with bfast v0.7.0a [66]. To analyse the genome content of hybrids, read depths (RD) were computed with bedtools v2.17.0 [67]. Mean RD in 10 kb sliding windows of 1 kb steps were calculated and plotted with ggplot2 [68].

An RD ratio was calculated for each gene shared between *Sc* and *Sk* based on the mean RDs for each gene in each subgenome, obtained as

$$R=\frac{\bar{RD_{Sc}}}{\left(\bar{RD_{Sc}}+\bar{RD_{Sk}}\right)}$$

This ratio goes from 0, when the *Sk* gene is the only present, to 1, when the only gene present is from *Sc*. Count histograms were plotted to calculate the average ratio in each hybrid genome, for this purpose large regions with ratios of 0 or 1, indicating that these regions were lost after the hybridization event, were excluded. This average ratio was considered as the expected hybrid ratio, i.e. the ratio between subgenomes in the ancestral hybrid of each strain just after the hybridization event. The expected hybrid ratio was then subtracted to each gene ratio to obtain the deviation from expectation. This deviation is positive or negative, if the *Sc* or the *Sk* subgenomes, respectively, increased after the hybridization event. We considered that a gene conserved its original hybrid state if its deviation was between 0.05 and −0.05, due to the noise observed. Again, due to noise, we used 0.1 and −0.1 as thresholds to consider that a gene effectively increased its *Sc* and *Sk* dosage, respectively.

### Phylogenetic and population genetic analyses

Each gene sequence was extracted from the annotation of the natural hybrids and classified as belonging to the *Sc* and *Sk* hybrid subgenomes. For each species, alignments were obtained with mafft v7.221 [69], for the translated amino acid sequences of orthologous genes from hybrids and four reference *Sk* strains or 75 *Sc* genomes representative of different clades and origins (Table S1). The aligned amino acid sequences were back-translated to nucleotides and the whole set of alignments concatenated. RAxML v8.1.24 [70] was used to construct a maximum likelihood (ML) phylogeny based on the concatenated alignment with model GTR-Γ and 100 bootstrap replicates. The concatenated alignments were also used to obtain a Neighbor-Net phylogenetic network based on the GTR-Γ corrected nucleotide distances with SplitsTree v4.14.6 [71]. Trees were drawn using iTOL v3 [72].

The individual-based Bayesian clustering method, implemented in structure v2.3.4 [73], was used to investigate population subdivisions with admixture to determine the origin of the *Sc* parents of hybrids. structure is based on the use of Markov chain Monte Carlo (MCMC) simulations to infer the assignment of genotypes into K distinct clusters (populations). The analysis was based on 10 000 randomly selected single nucleotide polymorphisms (SNPs) extracted from the *Sc* concatenated alignment. Five independent analyses were carried out for each number of clusters *K* (2≤*K*≤12). We determined the amount of additional information explained by increasing *K* using the Δ*K* statistic [74] with the program structure harvester version v0.6.94 [75]. Repetitions were then merged with clumpp v1.1.2 [76], and the results plotted with structure plot v.2.0 [77].

To determine the putative origin of the *Sc* subgenome in the hybrid MR25, we calculated for each gene the *p* distance (nucleotide substitutions per nucleotide site) between MR25 and the closest wine and beer2 strains. Then, we estimated for each gene the log_10_ of the ratio between the distance beer2-MR25, corrected by the average distance beer2-MR25 for all genes, and the distance wine-MR25, also corrected by the average distance wine-MR25 for all genes. Corrections were performed due to the intraspecific hybrid nature of the *Sc* beer strains [78]. Genes were ordered according to their genome position in the reference strain and the log_10_ ratio of the corrected distances were plotted using ggplot2 in R.

### Sporulation assays

Yeast cells were incubated on acetate medium (1 % sodium acetate, 0.1 % glucose, 0.125 % yeast extract and 2 % agar) for 5–7 days at 28 °C to induce sporulation. In total, 16 asci were collected for each strain when they were present. Ascus wall was digested with β 1,3-glucuronidase (Sigma) adjusted to 2 mg ml^−1^, and spores were then dissected in GPY agar plates with a Singer MSM manual micromanipulator. Spores were incubated at 28 °C for 3–5 days, and then, their viability was tested.

### *MAT* locus analysis

DNA from each hybrid strain was extracted according to Querol *et al*. [53]. The *MAT* locus was amplified with the same ‘MATa’ (5′-ACTCCACTTCAAGTAAGAGTTTG-3′) and ‘MATalpha’ (5′-GCACGGAATATGGGACTACTTCG-3′) specific primers described for *Sc* by Huxley *et al*. [79], but with a modified ‘MAT common’ primer (5′-AGTCACATCAAGRTCGTTYATG-3′) to also allow the amplification of the *MAT* locus of *Sk*. PCR reactions were performed in 100 µl final volume following the NZYTAqII DNA polymerase supplier instructions, under the following conditions: initial denaturing at 94 °C for 5 min, then 30 PCR cycles with the following steps: denaturing at 94 °C for 30 s, annealing at 58 °C for 30 s and extension at 72 °C for 30 s; and a final extension at 72 °C for 7 min.

The *Sc* and *Sk MAT* locus were differentiated by restriction analysis with endonuclease *Mse*I (Figure S1). Simple digestions of the PCR products with *Ms*eI (FastDigest SaqAI, ThermoScientific) were performed with 15 µl of amplified DNA to a final volume of 20 µl at 37 °C according to the supplier’s instructions. Restriction fragments were separated on 3 % agarose gel in 0.5× TBE buffer and a mixture of 50 bp 100 bp DNA ladder markers (Roche Molecular Biochemicals, Mannheim, Germany) served as size standards.

### Artificial *S. cerevisiae* x *S. kudriavzevii* hybrids obtained by rare-mating

Artificial hybrids were generated by rare-mating between the diploid wine *Sc* T73 strain and the diploid wild European *Sk* CR85 strain. As mentioned, genome sequences of these strains are available from previous studies [54, 55]. Antibiotic resistances were used as hybrid selection markers. For this purpose, strains T73 and CR85 were transformed with geneticin G418-resistance pGREG526 [80] and hygromycin B-resistance pRS41H [81] centromeric plasmids, respectively, by using the LiAc/SS carrier DNA/PEG method [82].

Rare-mating was performed according to Spencer and Spencer [83], with slight modifications [27]. Strains carrying the resistance plasmids were grown separately in 25 ml GPY broth with 200 µg ml^−1^ of the corresponding antibiotic for 48 h at 25 °C. Cells were recovered by centrifugation (3000 ***g*** for 5 min at room temperature), and the pairs of yeast cultures to be hybridized were placed together in the same tube. Aliquots of these mixed strains were inoculated in 20 ml of fresh GPY medium. After 5–10 days of static incubation in the slanted position at 25 °C, cells were recovered by centrifugation (3000 ***g*** for 5 min at room temperature), washed in sterile water, re-suspended in 1 ml of PBS and incubated for 2 h. A heavy suspension of the mixed culture was spread on GPY plates supplemented with 200 µg ml^−1^ of each antibiotic and incubated at 25 °C for 48 h. Colonies, resistant to both antibiotics, were isolated and purified by re-streaking on the same medium (GPY with both antibiotics). The hybrid nature of these colonies was confirmed by PCR amplification of the *BRE5* and *PPR1* protein-encoding nuclear genes, and the subsequent RFLP analysis with restriction enzyme *Hae*III (Takara Bio) as described elsewhere [84].

## Results

### Phylogeny reveals several independent hybridization events

In this study, we sequenced the genome of seven *Sc* x *Sk* natural hybrids (Table 1) to decipher their origins and mating process. One of the first questions is whether these natural hybrids are the result of one single hybridization event followed by diversification or are derived from independent hybridization events. We assembled and annotated the genome of the hybrids and extracted the coding sequence of each gene to reconstruct a multi-locus phylogeny for each of the subgenomes.

**Fig. 1.**
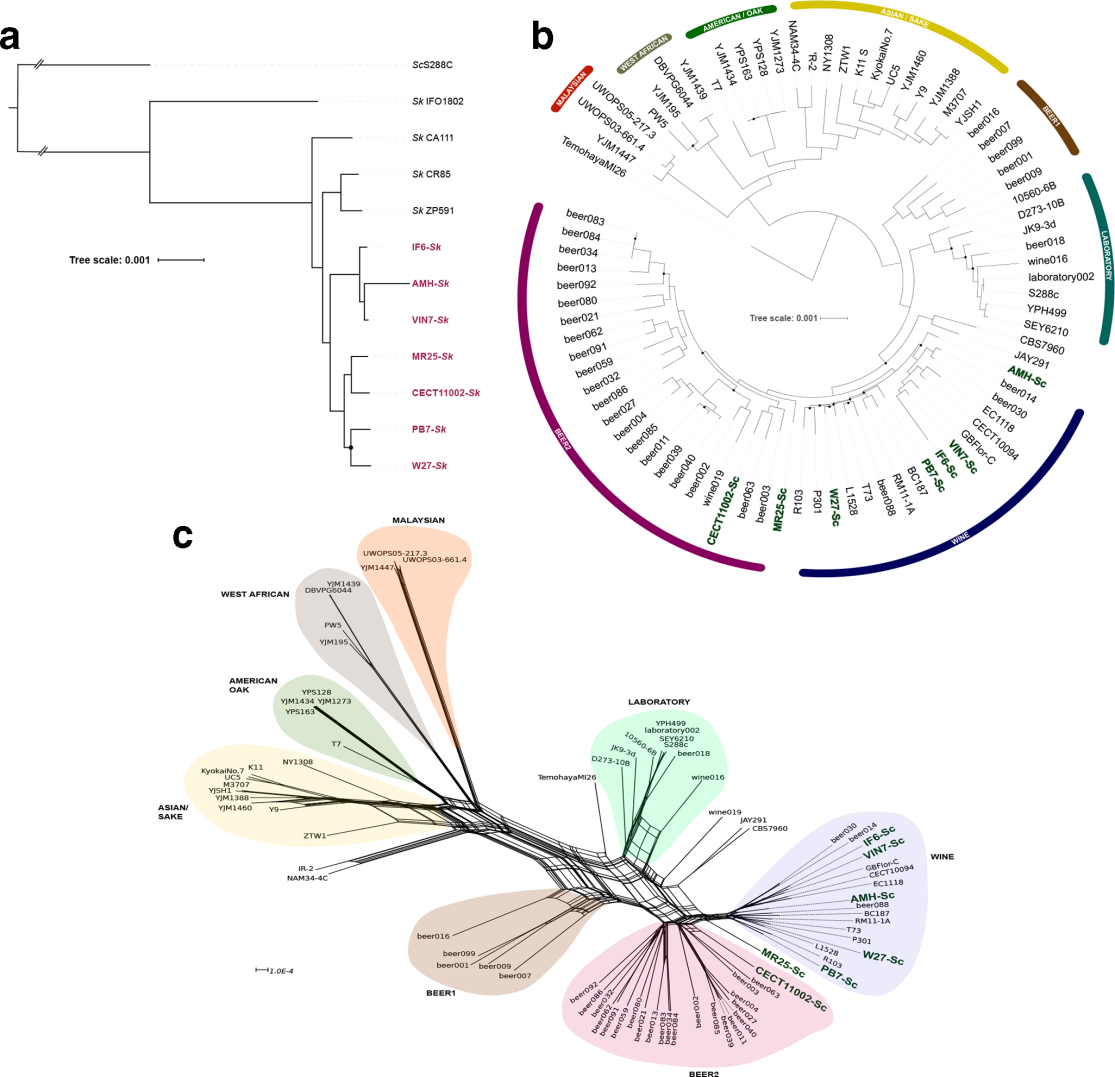
Phylogenetic analysis of the natural hybrids genomes. (a) ML tree of 647 concatenated genes alignment with four *Sk* strains available and *Sc* S288C as outgroup. (b) In total, 75 *Sc* strains representatives of different groups and 538 genes were used to reconstruct an ML phylogeny of the *Sc* strains and *Sc* subgenome of the hybrids. Black dots represent nodes with bootstrap values <0.70. (c) The same alignment used in (b) was also used to perform a Neighbor-Net analysis.

**Fig. 2.**
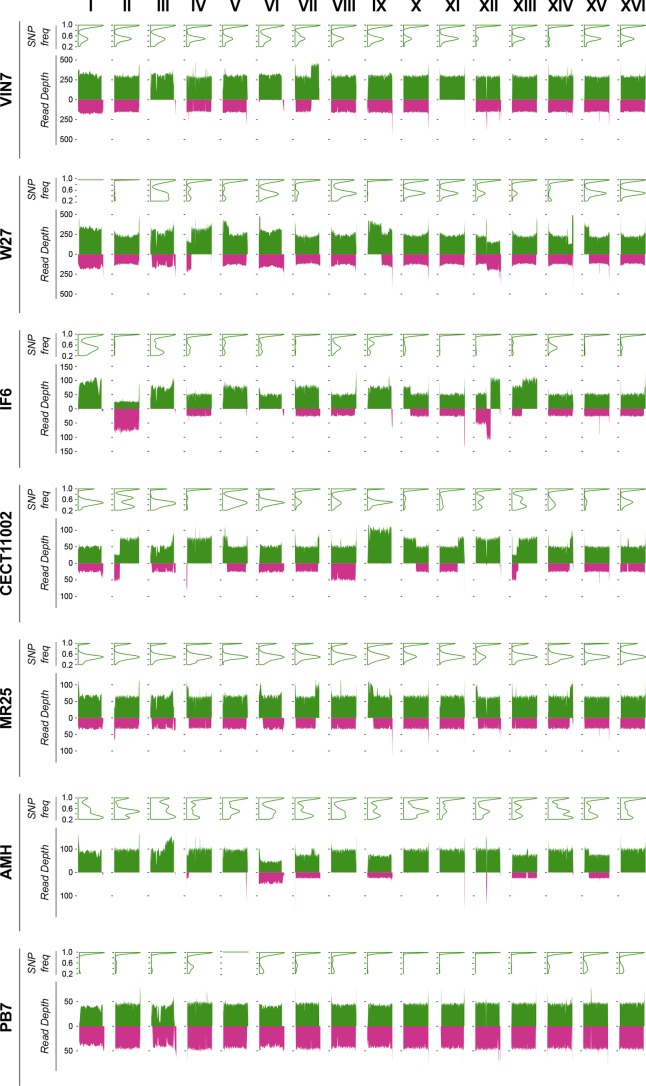
Genome composition of the strains. For each strain, we represent (up) the SNP frequency-density distribution in *Sc* subgenome and (down) the RD for each chromosome. The SNP frequencies are represented in the *y*-axis and the density is represented for the whole chromosome in the *Sc* subgenome (SNP distribution along the chromosome is shown in Fig. S5). Most of the chromosomes show two peaks, one around 1 that are homozygous SNPs, and a second one around 0.5, heterozygous SNPs in the strains that have two *Sc* copies. This distribution changes according to the ploidy or presence of aneuploidies. RD is represented for 10 kb windows moving by 1000 nt. The *Sc* subgenome is coloured in green and *Sk* subgenome in purple.

In the reconstruction of the phylogenetic history of the *Sk* genome fraction of the hybrids, we only used 647 genes common to all hybrids and the four *Sk* genomes currently available. This low number of genes is due to the extreme reduction of the *Sk* subgenome in the AMH strain (see Fig. 2). In the *Sk* subgenome ML phylogeny (Fig. 1a) and Neighbor-Net phylogenetic network (Fig. S2), hybrids cluster together as a sister group closely related to the reference European *Sk* strains from Spain (CA111 and CR85) and Portugal (ZP591). Despite the low number of strains available, we can observe that hybrids conform three different subgroups: one including IF6, VIN7 and AMH, a second comprising MR25 and CECT11002, and a third possible subgroup formed by PB7 and W27, although not significant according to its bootstrap value (68%). These results indicate that hybridizations involved several European *Sk* strains, closely related but different from the Iberian strains.

To investigate the origin of the *Sc* subgenome, we used 75 *Sc* strains, representative of different groups described in previous studies on the *Sc* population genomic diversity [35, 85–87]. In this case, 538 orthologous gene alignments were concatenated to obtain an ML phylogeny (Fig. 1b), a Neighbor-Net phylogenetic network (Fig. 1c), and a Bayesian population structure analysis (Fig. S3), all of them reproducing similar *Sc* populations.

Wine hybrids clearly cluster within the wine population in two separated subgroups: the typical wine strains, which include PB7 and W27, and the biofilm-forming *flor* strains, comprising hybrids VIN7, IF6 and AMH. Interestingly, IF6, which was isolated from a dietetic complement, belongs to the *flor* clade and is closely related to VIN7. The clinical isolate MR25 appears in an independent lineage, not included in any of the groups considered. This isolate could be an admixed strain as it appears in the Neighbor-net phylogenetic network in an intermediate position between wine and Beer2 populations (Fig. 1b and c) and appears as an admixed strain according to both the Bayesian population structure analysis (Fig. S3) and the analysis of genome admixture to visualize which population, beer2 or wine, is closest to each gene of the MR25 *Sc* subgenome (Fig. S4). Finally, the brewing hybrid CECT11002 clusters within the Beer2 group (Fig. 1b and c), together with the admixed brewing *Sc* strains (Fig. S3). In general, hybrid *Sc* subgenomes clustered according to their isolation sources, a result supporting independent hybridization events in different locations and environments.

### Hybridizations mainly involved, but not only, rare-mating as the main conjugation mechanism

Yeasts from the *Saccharomyces* genus usually conjugate by ‘canonical’ mating [88] between haploid cells/spores of opposite mating types, a and α, either from the same tetrad (automixis), from different asci (amphimixis), or derived from a mating-type switch (haploselfing), with different genetic consequences [89, 90]. In all cases, the resulting cells of ‘canonical’ crosses are diploid and heterozygous for mating types, which lack the mating ability (non-maters). However, these diploid non-maters can also conjugate by ‘rare’ mating [91], when they become mating competent by a mating-type conversion to a homozygous genotype [92]. Consequently, hybrid genomic architectures will differ depending on which conjugation type was involved in the hybridization events. To unveil the mating mechanisms involved in the generation of these hybrids, we genetically characterized our hybrids by using read mapping and flow cytometry, to determine their genome compositions and ploidies, as well as variant calling analysis, to measure their levels of heterozygosity. In addition, we also measured sporulation capability and spore viability in most hybrids (Table 1).

The ploidy data shows that most of the hybrids are allotriploids, with the exception of AMH and PB7, which are allotetraploids (Table 1). The contribution of each parental species to the hybrid genomes is shown in Fig. 2. In the allotriploid hybrids, we observe that the *Sc* content is twice that of *Sk*, in most parts of the genome. Hybrids VIN7, W27, IF6, CECT11002 and MR25 are triploid with a diploid contribution of *Sc* and haploid contribution of *Sk*. In addition, they also present different aneuploidies (polysomies and monosomies), chimeric chromosomes due to recombination between *Sc* and *Sk* homeologous chromosomes, or loss of certain non-centromeric chromosomal regions (see next section). Low spore viabilities shown by these hybrids, ranging from 0 to 11 % (Table 1), are also in accordance with their allotriploid nature.

As the hybrid *Sc* subgenomes are diploid, we assessed heterozygosity levels by constructing a density plot of the SNP frequency for each chromosome (Fig. S5). Interestingly, the *Sc* subgenome of the triploid hybrids showed different levels of heterozygosity according to the strain. Thus, wine strains VIN7, W27 an IF6 exhibit between 5000 and 2800 heterozygous SNPs (Table S2 and Fig. S5), which is an intermediate heterozygosity level, typical of wine strains [35, 85]. In contrast, the brewing CECT11002 and the clinical MR25 hybrids show higher levels of heterozygosity, 10 969 and 16 468 heterozygous SNPs, respectively. These higher levels are in accordance with the putative brewing origin of their *Sc* parents, which are typical among the beer *Sc* strains [85], due to their admixed origins [78]. The genome architecture of the allotriploid hybrids and their levels of heterozygosity can only be explained if hybrids were originated by rare-mating crosses between haploid *Sk* cells or spores and mating-competent heterozygous diploid *Sc* strains of wine or beer origins, depending on the hybrid.

As mentioned before, two exceptions to triploidy are hybrids AMH and PB7, which differ from others, and among them, in their genome compositions (Fig. 2). On one hand, AMH is a tetraploid that shows an extreme reduction of the *Sk* subgenome contribution. Most AMH genome consists of four copies of *Sc* chromosomes, except chromosomes VI, VII, IX, XIII and XV, with three *Sc* copies and one *Sk*, and chromosome IV, with three *Sc* copies and a chimerical copy with a tiny part of the left arm of *Sk*. This AMH hybrid appears as sterile, unable to sporulate (Table 1), which is likely due to the wrong segregation of the *Sc* tetravalent during meiosis.

Interestingly, the AMH *Sc* subgenome is highly heterozygous, and its SNP frequencies are compatible with the tri- or tetrasomy of its *Sc* chromosomes. The fact that the AMH tetraploid genome is mainly coming from *Sc*, together with the extreme reduction of the *Sk* haploid contribution, could be explained by two consecutive hybridization events. One possibility is that an allotriploid hybrid, originated by a similar mechanism than the other wine allotriploids, later conjugated by rare-mating with a haploid *Sc* to form this tetraploid, with a subsequent drastic *Sk* subgenome reduction due to genome instability. An alternative explanation is based on the observation that hybrids can generate viable spores (<1–5 %) because they contain most of their chromosomes coming from the same parent. Therefore, one of these rarely viable spores, carrying two copies of most *Sc* chromosomes and one copy of few *Sk* chromosomes, conjugated with a mating-competent diploid *S. cerevisiae* cell to generate the AMH hybrid.

On the other hand, PB7 is a perfect allotetraeuploid with two complete homozygous copies of each parental subgenome. As its *Sc* subgenome is coming from a parental wine strain, moderate heterozygosity would be expected, however, the few heterozygous SNPs are located in subtelomeric repetitive regions (Fig. S5). Therefore, the most probable mechanism that originated this allotetraeuploid hybrid, with two homozygous copies of each subgenome, is a spore-to-spore conjugation followed by a subsequent WGD to become an amphidiploid with spore viability of 95.3 % (Table 1).

#### Homeologous recombination drives genome evolution and reduces fertility

An interesting and open question is to understand how the hybrid genome content evolved after hybridization. Here we used a simple ratio between *Sc* and *Sk* gene contents and its deviation from the expected ratio to untangle which changes occurred in the different hybrid genomes. We calculated for each gene of the hybrid genome a RD ratio (see Methods). This ratio goes from 0, when only the *Sk* allele is present, to 1, when only the *Sc* allele is present. We used histograms of the frequencies of RD ratios to determine the average RD ratio for each strain, which was then subtracted to each gene RD ratio to calculate the deviation from the hybrid-state expectation.

**Fig. 3.**
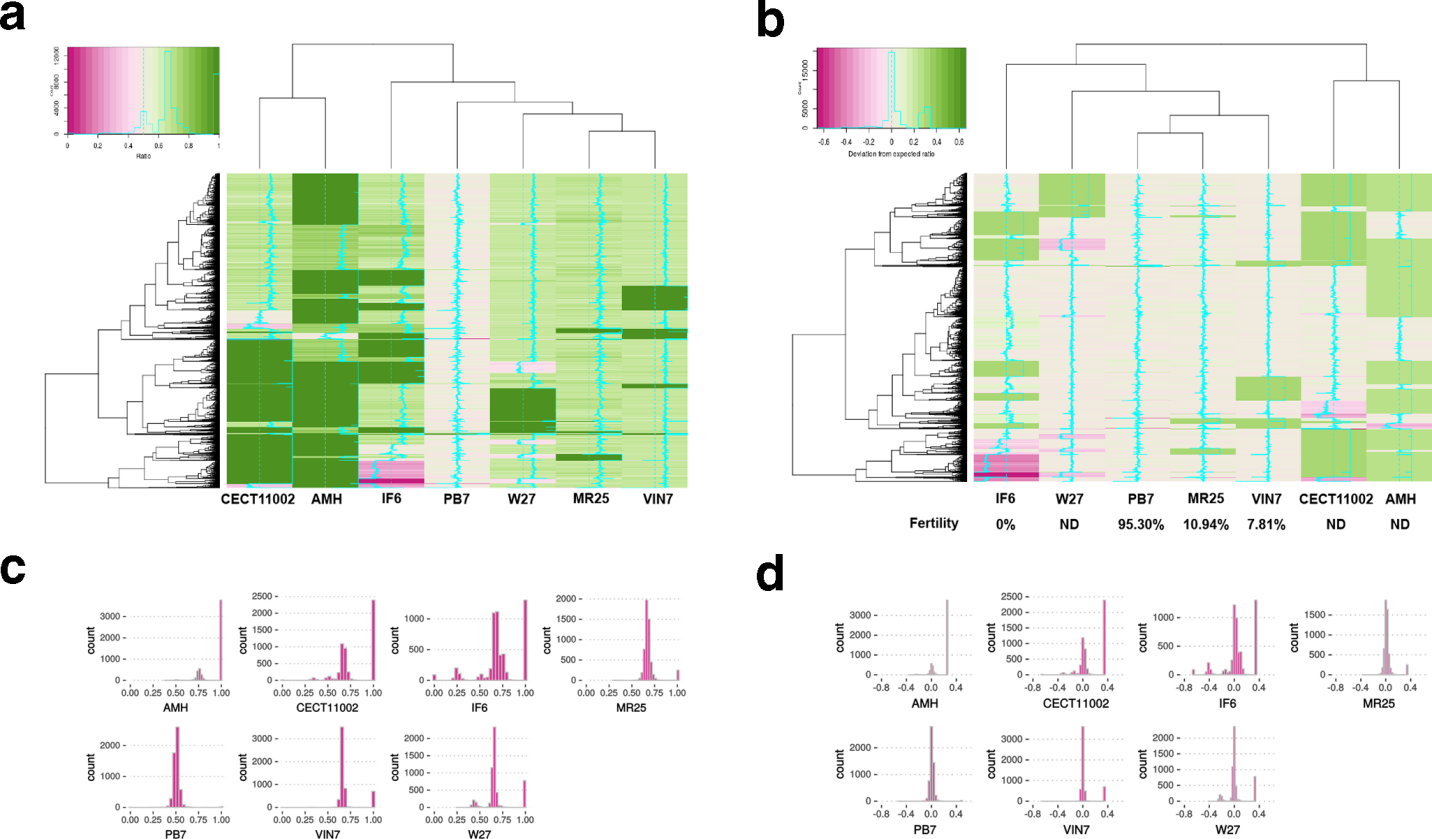
For each gene, we calculated a ratio of the genomic content of *Sc* vs *Sk*. (a) Heatmap of the ratio of *Sc*/*Sk*. The values go from 0 if only *Sk* alleles are present (purple) to 1 if only *Sc* is present (green). (c) Histogram of the count of each different ratio used to calculate the most common hybrid constitution. Most hybrids have 0.66 as the most common peak except AMH (0.75) and PB7 (0.5). (b) Heatmap of the deviation from the expected hybrid ratio [observed in (c)]. Values are negatives if the change is to increase *Sk* (purple) and positive if the change is to increase *Sc* (green). Fertilities of the different strains are shown under each strain (Table 1). (d) Histogram of the count of the deviation from the expected ratio showing the directional replacement to *Sc* in most of the hybrids.

For this purpose, we analysed a total of 5449 genes, that were present and annotated in both reference strains *Sc* T73 and *Sk* CR85 (Table S3). The RD ratio clearly shows that the *Sc* subgenome content is higher in all hybrids except PB7 (Fig. 3a). We established the expected ratio for each strain from the most frequent RD ratio (Fig. 3c), excluding ratios of 0 or 1, indicative of the loss of the *Sc* or *Sk* allele, respectively. Triploid strains MR25, W27, VIN7 and CECT11002 had a most frequent ratio of 0.66, congruent with their origins from hybridization events between diploid *Sc* and haploid *Sk* cells. Although the triploid IF6 hybrid shows a wider range of RD ratios due to aneuploidies, the expected hybrid ratio is 0.66, indicating a similar origin than the other triploid hybrids. In the tetraploid AMH, the most frequent hybrid ratio is 0.75, which confirms its 3 n *Sc* and n *Sk* contributions. Finally, PB7 showed the less diverse ratio distribution with an expected hybrid ratio of 0.5, indicating an equal contribution of *Sc* and *Sk* to its origin.

The deviation from the expected ratio (Fig. 3d) shows that most changes imply replacements of the *Sk* alleles by *Sc*. Thus, IF6 shows the most important number of changes, which could indicate a higher genomic instability. On the other hand, PB7 shows few changes being an almost perfect hybrid.

It is interesting to remark that only 290 genes maintain their original hybrid ratio in all strains, and most of them, 284, are located on chromosome XV, indicating that this chromosome is the only that preserved its original *Sc/Sk* proportion in all hybrids. In general, ratio deviations observed are grouped in blocks of genes located in the same chromosome regions, which indicates that chromosome loss and the generation of chromosome chimeras due to recombination between homeologous chromosomes are the main mechanisms involved in changes of the genome composition of hybrids, usually biased towards a reduction of the *Sk* genome fractions. However, most chromosome rearrangements are specific of each hybrid, and very few are shared between the different strains. In fact, only six genes show a replacement in all hybrids, including PB7, of the *Sk* allele by the *Sc* one. These are *BUD5*, which is overlapping the *MAT* locus on the opposite strand of chromosome III; *EFT2*, located on chromosome IV and encoding a translation elongation paralogous to *EFT1*; and a subtelomeric region encompassing genes *FZF1*, *ZRT1*, *ADH4* and *MNT2*, which is located on the left arm of chromosome VII. This region corresponds to the only homeologous recombination shared by all hybrid strains.

Interestingly, the gene *BUD5*, which overlaps the X region of the *MAT* locus, came from *Sc* in all natural hybrids. We performed a PCR amplification and subsequent restriction analysis to determine the parental origins of the *MAT* locus *a* and *alpha* idiotypes present in hybrids. In all cases, both idiotypes came from *Sc*, and no *Sk* idiotype is present (Fig. S1). This seems to be incongruent with both a rare-mating or a spore-to-spore canonical mating as the origin of hybrids. However, only opposing mating types can mate, which suggests that the *Sk MAT* idiotype was replaced after the hybridization event by the homologous idiotype from *Sc*, perhaps due to an incompatibility between *MAT* loci from different species in the long term. This is only possible by HO-mediated recombination with the HML or HMR silent cassettes.

Finally, there seems to be a correspondence between the number of genome rearrangements and hybrids' fertility. Thus, MR25 and VIN7 are clustered together with PB7 in the ratio deviation analysis (Fig. 3b) because they exhibit the smallest number of chromosomal rearrangements, and interestingly are the allotriploids with highest fertilities, with spore viabilities of 10.9 and 7.8 %, respectively (Table 1). The other allotriploids exhibit more genome rearrangements and their spore viabilities are 0%, or no asci were detected. Therefore, the higher the number of homeologous chromosome rearrangements, the lower the spore viability.

#### Artificial hybridization by rare-mating reproduces the genome architecture of natural hybrids

Artificial hybridization is increasingly used to improve industrial *Saccharomyces* yeasts [27, 49]. One of the methods used to generate artificial hybrids is rare-mating because they acquire the whole genome of both parents to combine most of their physiological properties.

**Fig. 4.**
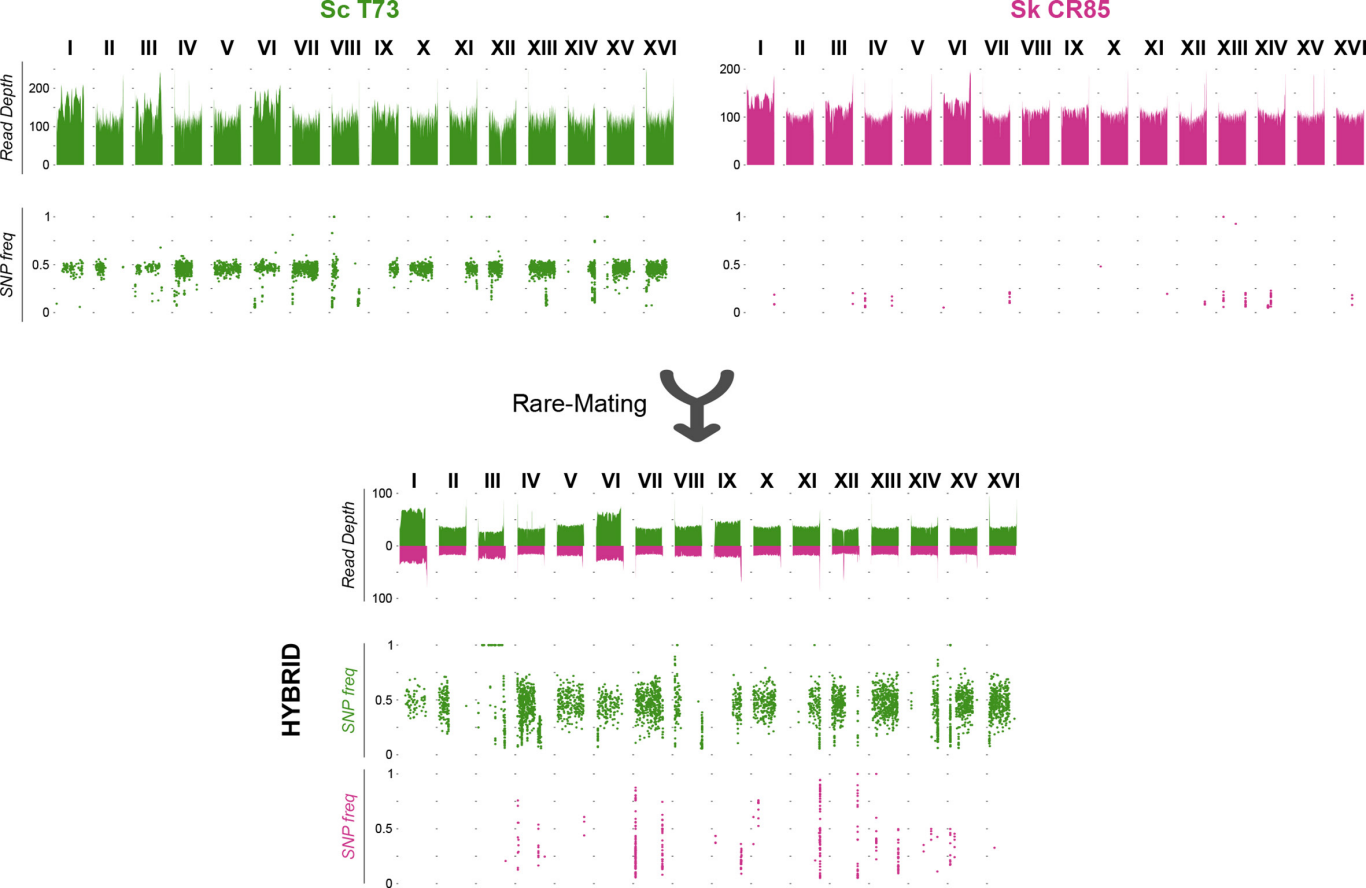
Genomes of the parental strains and the artificial hybrid obtained by rare-mating. *Sc* and *Sc* subgenome of the hybrids are represented in green, and *Sk*, in purple. The RD and SNP frequencies are represented along the genome. The *Sc* T73 is a diploid heterozygous wine strain with several LOH events in different chromosomes. *Sk* CR85 is also diploid but homozygous. The resulting hybrid has two copies of the *Sc* subgenome and one of the *Sk* subgenome and retains the heterozygosity with the LOH events of the *Sc* parental. Chromosome III only has one copy of *Sc* and one of *Sk* as confirms the LOH in the whole chromosome.

As seen, our study indicates that most natural hybrids were also generated by rare-mating. Therefore, we decided to replicate a natural hybridization in the lab by a ‘rate-mate’ crossing of the diploid wine *Sc* strain T73, closely related to the *Sc* parental, and the diploid *Sk* CR85, isolated from an oak tree in Spain. For hybrid selection, parental strains were transformed with antibiotic-resistance plasmids (see Methods), which are easily removed after hybridization, to avoid the effect of the use of auxotrophic mutations. Once an artificial hybrid was obtained, its genome was sequenced, assembled and compared with the genomes of the parental strains (Fig. 4), whose sequences were available [54, 55].

The parental *Sc* T73 possesses a diploid genome with a moderate heterozygosity level, as expected for a wine strain, and several homozygous regions due to loss of heterozygosity events. The parental *Sk* CR85 genome is diploid and completely homozygous, which is common in natural *Saccharomyces* strains, due to regular haploselfing events. The resulting hybrid is an allotriploid (3,18±0.01) with two copies of the *Sc* genome fraction, confirmed by the heterozygous SNP frequencies, and one copy of the *Sk* subgenome. Interestingly, the only exception is chromosome III, in which the *MAT* locus is located. RD in the hybrid genome (Fig. 4) shows that it contains one single chromosome III copy from *Sc* and another from *Sk*. This is confirmed by the analysis of heterozygosity in the *Sc* subgenome. In the other *Sc* chromosomes, levels of heterozygosity are identical to those found in the parental *Sc* chromosomes. However, in the artificial hybrid, the levels of heterozygosity of *Sc* chromosome III drop to 0, in accordance with the presence of one single copy. This result indicates that the parental diploid *Sc* T73 cell, involved in the hybridization event, acquired mating-competence not by becoming homozygous for the *MAT* locus due to gene conversion, but by becoming hemizygous for the *MAT* locus due to a chromosome III copy loss (monosomy). This is congruent with the fact that chromosome III is one of the smallest chromosomes and shows the highest loss frequency in *S. cerevisiae* [93].

As the original *Sk* parent was diploid and the hybrid only contains one copy of the *Sk* genome fraction, two hypotheses could explain how this genome composition was generated in the hybrid. In the first hypothesis, the hybrid originated by a ‘rare-mating’ between two competent diploid cells and an immediate loss of one complete copy of the *Sk* subgenome after mating. The second involves a rare-mating between a competent diploid *Sc* cell and a haploid *Sk* spore/cell, which would require that *Sk* sporulation occurred in the rare-mating medium. To test whether *Sk* sporulation is possible under these conditions, we performed a ‘rare-mating’ experiment but only with *Sk* CR85, and after 5 days this culture was completely sporulated, which confirmed that the second hypothesis is the most probable.

It is worth noticing that the artificial hybrid contains the *Sc* MATa and *Sk* MATalpha idiotypes in the *MAT* locus. This different *MAT* locus composition between natural and artificial hybrids reinforces the hypothesis that an incompatibility exists between *MAT* loci from different species in the long term. Additional studies are necessary to understand how and why the Sc *MAT* locus is favoured in natural hybrids and if this is a more general trend in other interspecific hybrids.

To sum up, we confirm that artificial hybridization in laboratory conditions reproduces the most frequent genome architecture observed in natural hybrids, although the mechanisms to generate mating-competent *Sc* diploid cells were different.

## Discussion

Hybridization between species has for years been an intriguing phenomenon for biologists. Due to the improvement in genome-sequencing technologies, its importance in plants [6], animals and fungi [94] diversity and evolution are becoming clearer [95]. Hybridization between different species of the *Saccharomyces* genus was first suspected and confirmed in the lager beer *S. pastorianus*, also known as *S. carlsbergensis* [96, 97], a hybrid between *Sc* and *Se*. Since then, multiple hybrids between different *Saccharomyces* species were found principally in human-related environments, but also introgressions from different species were found in strains from natural habitats [14, 15]. The clear ability of *Saccharomyces* yeasts to form viable hybrids makes them an interesting model for hybridization studies. Here we investigated the genome of different *Sc* x *Sk* hybrids from different isolation sources to decipher the mechanisms used to mate and the evolution of their genomes.

A first question to ask is if the different hybrids came from a unique hybridization event or multiple events have occurred. Previous work on *Sc* x *Sk* hybrids using six genes pointed out that there were different events that gave birth to these hybrids [52]. Here we have used the whole genome of representatives of the different groups described by Peris *et al*. [52] and we have clearly seen that the hybrids came from different hybridization events. The *Sk* subgenome showed that three groups could exist. This result is consistent with those obtained in the *Sc* subgenome phylogenetic analysis. Thus, VIN7, IF6 and AMH clustered together with the *flor Sc* strains, but PB7 and W27 are more related to other wine strain lineages, which clearly indicate that they derived from different hybridization events. This is also supported by the fact that the mating mechanisms involved in their origins differ (see below). Interestingly, the beer strain CECT11002 is clustered within the beer2 or mosaic beer group. Lager brewing yeasts are *Sc* x *Se* hybrids and their *Sc* subgenomes cluster with the ale strains from the beer1 group [98]. The brewing *Sc* x *Se* hybrids could derive from a single *Sc* ancestor because all of them cluster together, but this ancestor was different from those generating the brewing *Sc* x *Sk* hybrids [98, 99].

**Fig. 5.**
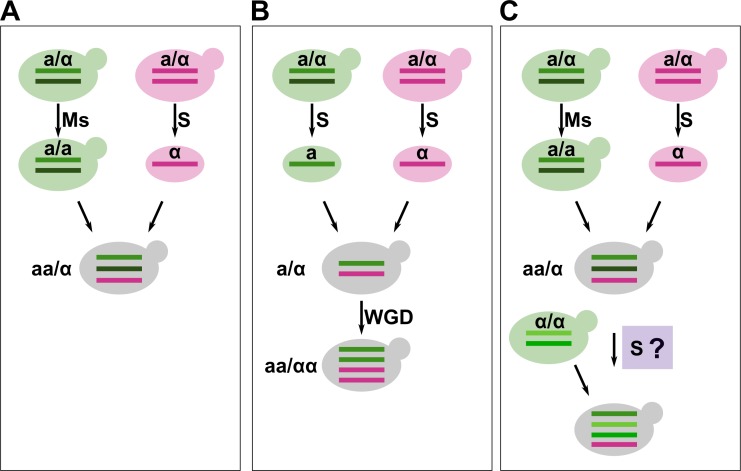
Models of the different mating mechanisms observed in the hybrids. The *Sc* parentals or spores are represented in green and the *Sk* in purple. Different shades of colours represent heterozygosity. *MAT* loci are assigned randomly and only as an example. Ms, *MAT* switch/loss of heterozygosity. S, sporulation. WGD, whole-genome duplication. (a) Rare-mating. A diploid *Sc* becomes competent to conjugate by the conversion of the *MAT* locus. The *Sk* parental sporulates. The competent diploid *Sc* mates with a *Sk* spore to form a triploid *Sc* x *Sk*. This is the mechanism used by most of the strains in the study. (b) Spore-to-spore cross and a subsequent whole-genome duplication, as observed in PB7. Both *Sc* and *Sk* sporulate, and spores mate to form a diploid hybrid. At some point, a whole-genome duplication occurs forming a tetraploid. (c) Model for the formation of the AMH strain. A first hybridization event by rare-mating occurred, as in (a). Subsequently, the hybrid could have been forced to sporulate and mate with another diploid *Sc* to form a tetraploid with an extremely low *Sk* contribution.

The *Sc* x *Sk* hybrids isolated and analysed so far are mostly triploids, with a diploid contribution of *Sc* and a haploid contribution of *Sk* [51, 52, 100–102]. This genome composition opened up the hypothesis that the mating mechanism used to hybridize was rare-mating but could not be completely confirmed [13, 100]. The heterozygosity levels that these strains show on the *Sc* subgenome is, therefore, clarifying this question. We observe that, in the triploid strains, the heterozygosity levels are related to the isolation source and with the cluster of strains it belongs to. It was previously described that different populations of *Sc* have different heterozygosity levels [35, 85, 103]. CECT11002 and MR25, which are related to beer strains, have similar heterozygosity levels as *Sc* beer strains. Wine hybrids have similar heterozygotic positions as wine *Sc* strains. Conserving these levels in the *Sc* subgenome supports that rare-mating was the hybridization mechanism used by these strains, as summarized in Fig. 5a. This genome constitution is the most abundant in the strains sequenced here but also in the strains previously studied [100, 101], which could also be evidence that rare-mating is the most common mating mechanism in natural *Sc* x *Sk* hybrids.

The artificial hybrid generated by rare-mating ended up with a genome constitution similar to the most typical spontaneous hybrids. We used plasmids with antibiotic resistance to avoid the use of auxotrophies that could select for different genome composition or chromosomal aneuploidies. It was astonishing to find out that the mechanism used to become competent to conjugation in *Sc* was the complete loss of one copy of the chromosome III, which does not seem to happen in natural hybrids. Chromosome III has a high-loss frequency in the laboratory [93]. Chromosome loss is probably more frequent in a population in laboratory conditions than the loss of heterozygosity in the *MAT* locus. In natural conditions, aneuploidies can be detrimental and therefore less frequent in the population than *MAT* homozygous cells. This could explain why the same phenomenon is not observed in natural hybrids. What clearly stands out is that heterozygosity from a 2 n wine *Sc* is conserved in the hybrid and that, in *Sk*, sporulation is possible even in high nutrient concentration media. These two processes together make hybridization between diploid *Sc* and haploid *Sk* more frequent than spore to spore in both laboratory and natural conditions.

Despite triploidy being the most usual ploidy in natural hybrids, some tetraploid strains have been found. Here we reported two tetraploid strains with two different stories. PB7 is tetraploid with a perfect diploid homozygotic *Sc* and *Sk* subgenomes. Moreover, it is a fertile hybrid. The extreme homozygosity in the *Sc* subgenome is not compatible with its wine origin. Such a trait can only be explained if the genome content was doubled after a spore-to-spore hybridization, as recapitulated in Fig. 5b. Another tetraploid and fertile hybrid was found between *Sc* and *Su*, the so-called S6U strain [104]. The strain EL1D4, an *Sc* x *Sk* hybrid, was also found to be tetraploid and homozygous in the *Sc* subgenome but its sporulation was not tested [100]. In both cases, it was postulated that the fertility and the homozygosity, respectively, were the result of autopolyploidization after the mating event [100, 104]. Genome doubling to restore fertility is common in plant hybrid speciation [105]. This mechanism was also responsible for fertility restoration in the yeast *Zygosaccharomyces parabailii*, a recently discovered fertile hybrid [18]. A recent study in the laboratory found that becoming allopolyploid and recovering fertility in *S. paradoxus* hybrids could happen in less than 400 cell divisions [106]. Here we show that, even if these are rare events, this phenomenon can happen in industrial environments in *Saccharomyces* genus.

The other tetraploid strain, AMH, has a completely different genome composition. In this case, the *Sc* subgenome shows higher heterozygosity than the rest of the wine strains due to the presence of polysomic chromosomes. The *Sk* subgenome is highly eroded, resulting in few regions of *Sk* remaining in the genome. AMH strain is a commercial strain. It is known that hybridization between different *Sc* strains was used to improve different traits. Some of these strains were not recognized as hybrids and some of them can even sporulate with low viability, as we see here with the strains MR25 and VIN7. Other commercial strains have a reduced *Sk* genome remaining in their genome, this is the case of the Maurivin EP2 [107]. We hypothesize that this can be the result of such an improvement program were a spore from a triploid hybrid was crossed with a diploid commercial *Sc* strain (summarized in Fig. 5c), which could explain slightly higher heterozygosity in the *Sc* subgenome. If a hybrid was sporulated, meiotic recombination could have drastically reduced the *Sk* subgenome and the resulting spores could have only some chromosomes from *Sk* explaining such an important reduction in the *Sk* content of AMH.

Hybridization between *Sc* and other *Saccharomyces* species in wines or beer is thought to be adaptive to low-temperature environments [41–46]. Nor *Sk* or *Se*, which are more cryotolerant species than *Sc*, were isolated from industrial environments except in the form of hybrids with *Sc* [50, 108]. The *Sc* x *Sk* hybrids show a wide range of ability to grow at low temperatures, but it was shown that the higher the *Sk* content, the better the growth at low temperatures [43]. In the hybrids studied here, we observe that recombination between homeologous chromosomes (homologous from different species) is an important contributor to genomic diversity. Most of the changes observed are replacements of the *Sk* part by its homologous *Sc* region that have arisen by mitotic recombination. Few of these regions are shared between strains, indicating a stochastic phenomenon and/or different selective pressures acting on them. This mechanism introduces phenotypic variation which could explain the differences observed between the strains [43]. Interestingly, our results also suggest that mitotic recombination reinforces the post-zygotic barrier, as the strains with a higher number of recombination events have no spore viability or, even, cannot sporulate. Genomic instability is one of the proposed mechanisms of post-zygotic isolation [109] but could be important to improve phenotypic variability and adapt to fluctuant environments at expenses of sexual reproduction, which is rare or absent in industrial environments [110]. Hybridization could, therefore, be an interesting strategy to improve adaptability in two ways: by generating heterosis, due to the differences between the two subgenomes, proteomes, metabolomes and interactomes, as well as by increasing the genome instability to generate variability.

Therefore, hybridization between *S. cerevisiae* and *S. kudriavzevii* is a recurrent strategy in industrial environments involving the fusion between the metabolic capabilities of the two species. *Saccharomyces* species can mate using different mating mechanisms, but rare-mating is the most commonly used. The mechanism used to mate determines the genomic structure of the hybrid and its evolutionary outcomes. The evolution of hybrid genomes is triggered by genomic instability and results in a wide diversity in genomic rearrangements. The stressful environmental conditions in industrial fermentations could make hybrid genomes to preserve those chromosome rearrangements of adaptive value [111]. Therefore, interactions between both parental genomes, proteomes and metabolomes, together with the harsh environmental conditions present during fermentation, determine the final composition of hybrid genomes. In the case of *S. cerevisiae×S. kudriavzevii* hybrids, their genomes are mainly characterized by the preservation of the *S. cerevisiae* subgenome and a progressive reduction of the *S. kudriavzevii* fraction.

## Data bibliography

Genome sequences of *Saccharomyces cerevisiae* and *S. kudriavzevii* reference strains were obtained in the following studies:

1. Gallone B., Steensels J., Prahl T., Soriaga L., Saels V. *et al*. Domestication and divergence of *Saccharomyces cerevisiae* beer yeasts. *Cell* 2016;166(6):1397-1410.e1316.

2. Legras J.-L., Galeote V., Bigey F., Camarasa C., Marsit S. *et al*. Adaptation of S. *cerevisiae* to fermented food environments reveals remarkable genome plasticity and the footprints of domestication. *Mol. Biol. Evol.* 2018;35(7):1712-1727.

3. Liti G., Carter D.M., Moses A.M., Warringer J., Parts L. *et al*. Population genomics of domestic and wild yeasts. *Nature* 2009;458:337-341.

4. Macías L.G., Morard M., Toft C., Barrio E. Comparative genomics between *Saccharomyces kudriavzevii* and *S. cerevisiae* applied to identify mechanisms involved in adaptation. *Front Genet.* 2019;10:187.

5. Morard M., Macías L.G., Adam A.C., Lairón-Peris M., Pérez-Torrado R. *et al*. Aneuploidy and ethanol tolerance in *Saccharomyces cerevisiae*. *Front Genet.*, 2019;10:82.

6. Peter J., De Chiara M., Friedrich A., Yue J.-X., Pflieger D. *et al*. Genome evolution across 1,011 *Saccharomyces cerevisiae* isolates. *Nature* 2018;556:339–344.

## Supplementary Data

Supplementary material 1

Supplementary material 2
