# Supplementary material for: Genome structure reveals the diversity of mating mechanisms in Saccharomyces cerevisiae x Saccharomyces kudriavzevii hybrids, and the genomic instability that promotes phenotypic diversity
Source: Microb Genom. 2020 Feb 17;6(3):e000333. doi: 10.1099/mgen.0.000333 (PMC7200066; doi:10.1099/mgen.0.000333)

**Supplementary Figure S1.** Agarose gel electrophoresis showing the *MAT* locus restriction patterns of the natural (references in black) and artificial *Sc x Sk* hybrids, and *Sc* and *Sk* reference strains (indicated in green and brown, respectively). PCR fragments were amplified with MATa (top, amplicon length 544 bp) and MATalpha (bottom, amplicon length 404 bp) specific primers and digested with endonuclease *Mse*I to differentiate the *Sc* and *Sk* MAT loci. The length of the diagnostic bands, specific of *S. cerevisiae* and *S. kudriavzevii*, are indicated in green and brown, respectively. Restrictions fragments were separated on 3% agarose gel in 0.5x TBE buffer and a mixture of 50-bp 100-bp DNA ladder markers was used as size standards.

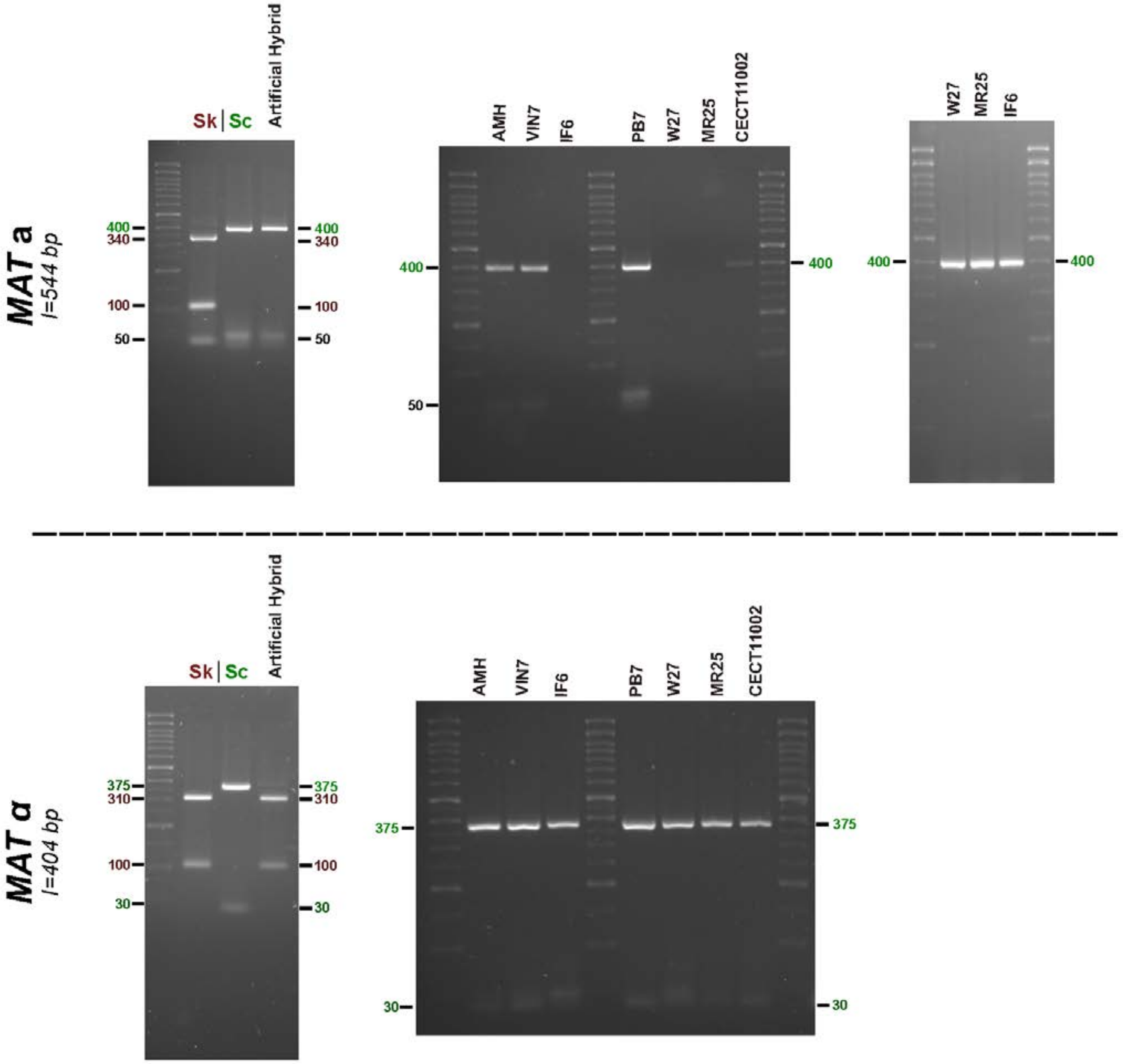

**Supplementary Figure S2.** Neighbor-Net phylogenetic network based on the GTR-Γ corrected nucleotide distances of the concatenated alignment of 647 genes common to the *Sk* subgenomes of natural *Sc* x *Sk* hybrids and four representative *Sk* strains.

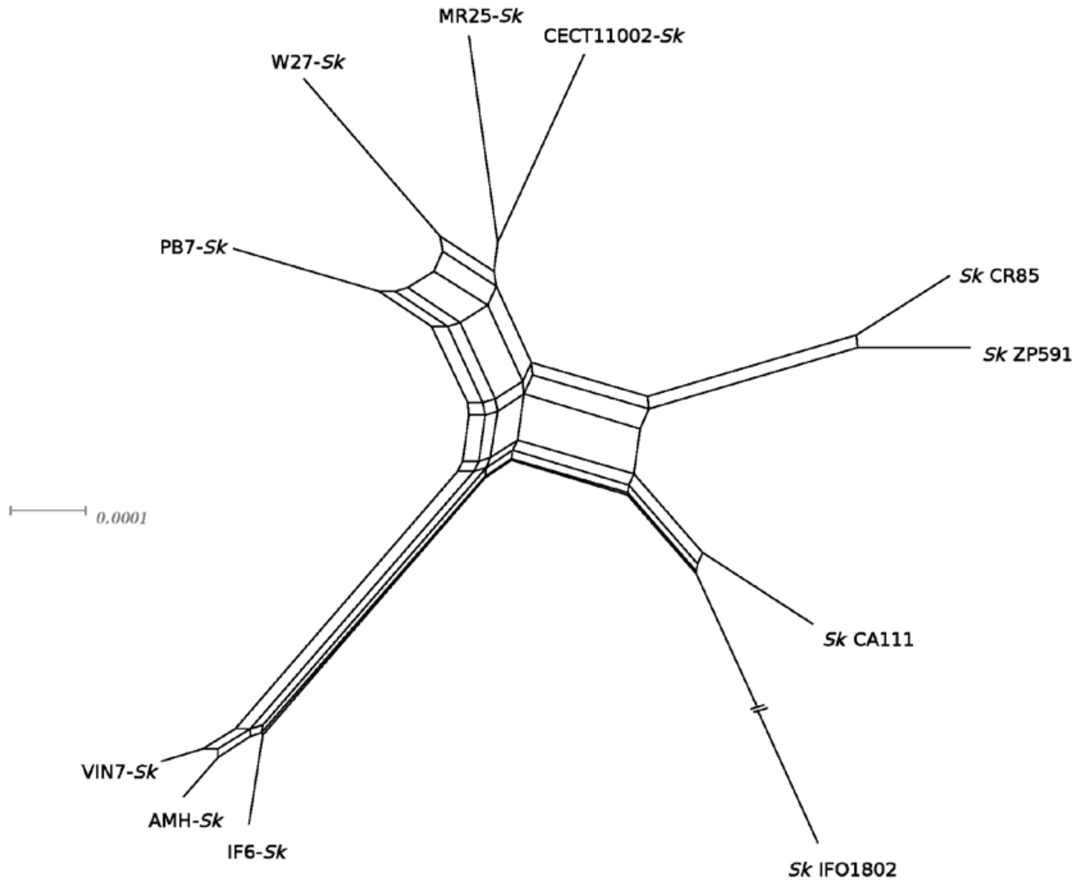

**Supplementary Figure S3.** Population structure analysis according to the individual-based Bayesian clustering method implemented in STRUCTURE, of the *Sc* subgenomes of hybrids (references in purple) and 75 *Sc* genomes (references in black) representative of different clades and origins (indicated at the lower part of the plot). The analysis was based 10000 randomly selected single nucleotide polymorphisms (SNPs) extracted from the *Sc* concatenated alignment of 538 orthologous genes. The most consistent number of genetic clusters/populations in this analysis was K = 6, according to the ΔK statistic. Each bar represents a particular strain, and bar colors represent population membership. The presence of several colors in the same bar (strain) suggests admixture.

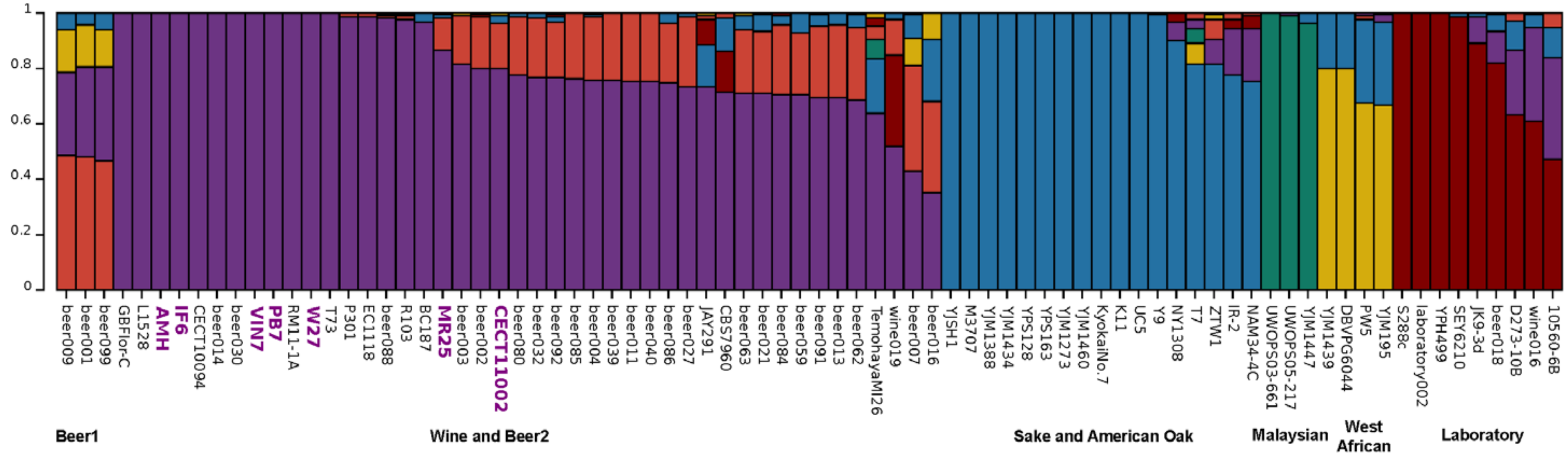

**Supplementary Figure S4.** Genome-wide analysis of admixture in the Sc subgenome of the hybrid strain MR25. To directly visualize which population, beer2 or wine, is closest to each region of the Sc subgenome of MR25, we calculated the log10 ratio of the corrected Beer2-MR25 nucleotide sequence divergence and the corrected minimum Beer2-MR25 nucleotide sequence divergence gene by gene along the genome. Log10 < 0 indicates which *Sc* genes of MR25 are more closely related to beer2 (orange and yellow lines, for even and odd chromosomes, respectively), and Log10 > 0 indicates which MR25 *Sc* genes are more closely related to wine (purple and green lines, for even and odd chromosomes, respectively). On the right side are indicated the mean distance of the MR25 *Sc* genes to beer 2 and wine, as well as the number (#) of genes closer to beer2, closer to wine, and those showing a similar distance to beer2 and wine ($d_B = d_W$).

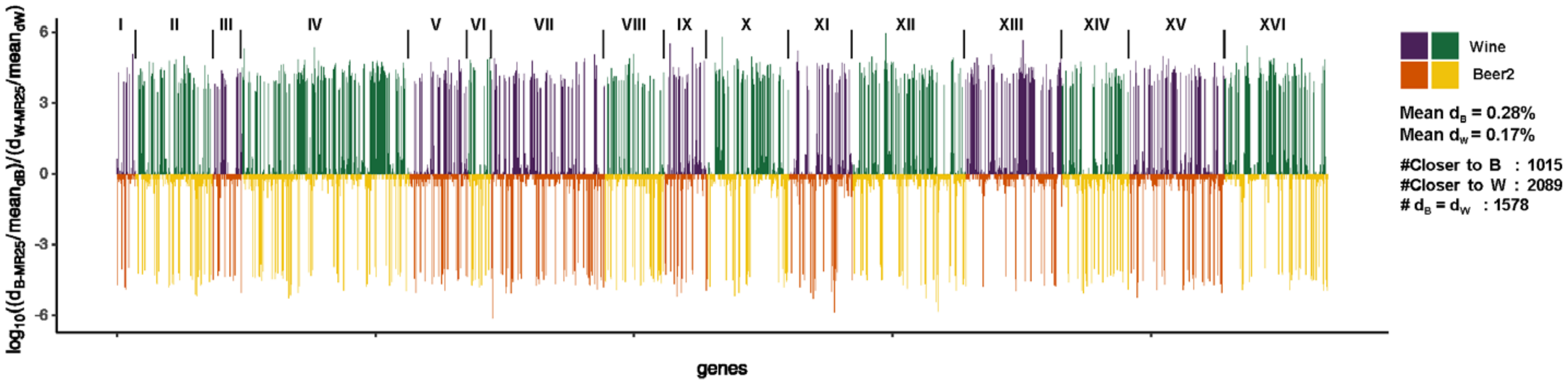

**Supplementary Figure S5.** Heterozygous SNP frequency distribution along the genome. Only Heterozygous SNPs are shown, in green for *Sc* and in purple for *Sk*. Frequencies are plotted along with the position of the SNPs in the chromosome.

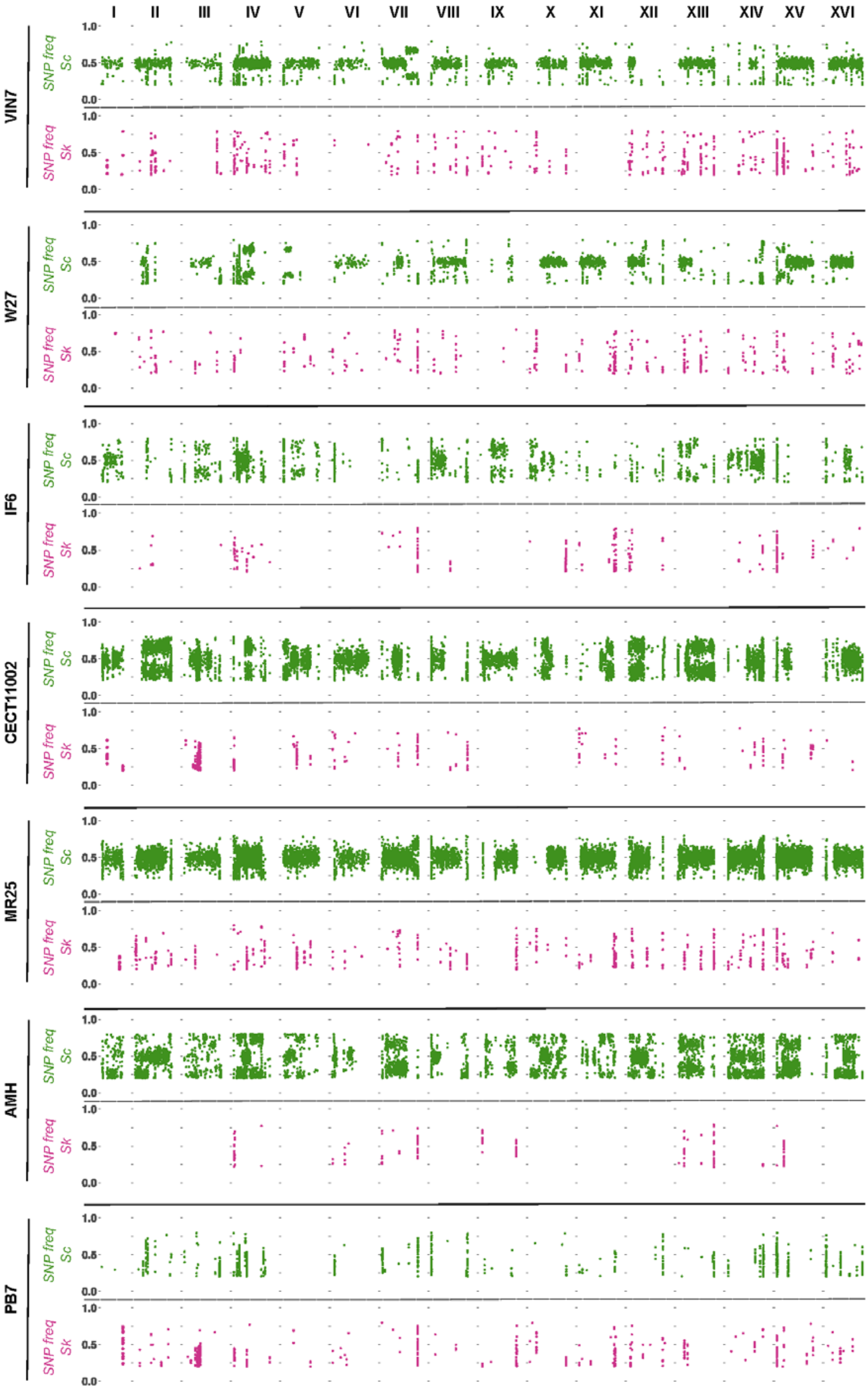

Supplement: Supplementary material 1 [file mgen-6-333-s001.pdf]
